# New Software for Gluten-Free Diet Evaluation and Nutritional Education

**DOI:** 10.3390/nu11102505

**Published:** 2019-10-17

**Authors:** Arrate Lasa, Idoia Larretxi, Edurne Simón, Itziar Churruca, Virginia Navarro, Olalla Martínez, María Ángeles Bustamante, Jonatan Miranda

**Affiliations:** Gluten Analysis Laboratory of the University of the Basque Country, Department of Nutrition and Food Science, University of the Basque Country (UPV/EHU), 01006 Vitoria, Spain; arrate.lasa@ehu.eus (A.L.); idoia.larrechi@ehu.eus (I.L.); edurne.simon@ehu.eus (E.S.); itziar.txurruka@ehu.eus (I.C.); olaia.martinez@ehu.eus (O.M.); jonatan.miranda@ehu.eus (J.M.)

**Keywords:** software, gluten-free diet, application, dietary balance, gluten-free product, nutritional education

## Abstract

Following a gluten-free diet (GFD) is the only treatment for celiac disease. This diet must ensure the absence of gluten but also needs to be nutritionally balanced. Dietitians working in this field cannot properly evaluate energy and nutrient intake of celiac people because dietary programs available on the market do not contain the nutritional composition of gluten-free products (GFP). Here we present a new GFD evaluation software that contains more than 700 gluten-free rendered foodstuffs and their macronutrient composition. Apart from diet evaluation and design, the software represents a tool for nutritional education as well, since it shows diet appropriacy and indicates how to promote balanced self-care. Moreover, anthropometric and biochemical data or symptoms presence and diet adherence can be recorded and evaluated. This open free software, can be downloaded in its app format for mobiles and tablets. Software evaluation indicated its correct functionality and the importance of assessing a GFD with GFP instead of with their gluten-containing analogues. Thus, this software represents an essential e-Health tool, not only for proper GFD evaluation, but also for improving life quality of celiac and gluten sensitive people.

## 1. Introduction

Celiac disease (CD) is an autoimmune enteropathy triggered by the ingestion of gluten-containing grains in susceptible individuals and is a common lifelong disorder worldwide [1]. Although the vast majority of those affected are undiagnosed, its prevalence in Europe is estimated at 1%. People with celiac disease suffer from intestinal (abdominal pain and distension, chronic diarrhea, etc.) and extra-intestinal symptoms (irritability, dermatitis herpetiformis, etc.) when ingesting gluten. Moreover, untreated CD can lead to long-term complications like osteoporosis, cancer, and autoimmune disorders [2].

The only effective treatment for CD is strict dietary gluten withdrawal, so people with celiac disease must control the presence of this protein in food. This means that they must avoid an important source of nutrients (some cereals, like wheat, barley, triticale, rye, and their derivatives), which can cause a significant imbalance in their diet. This disequilibrium can lead to the appearance of nutrient deficits, such as of iron, biotin, or folate, and may increase the risk of developing pathologies like anemia, diabetes, cardiovascular diseases, or osteoporosis [3,4,5,6,7,8]. While CD is the most common disorder triggered by gluten, other conditions also benefit from following this dietary restriction, such as Non-celiac Gluten Sensitivity, Dermatitis Herpetiformis, or Inflammatory Bowel Syndrome [2].

Besides the exclusion of gluten-containing natural products (wheat, barley, triticale, and rye), part of the dietetic strategy of those groups is to replace gluten-containing products with their gluten-free counterparts. However, their nutritional composition is not identical. In fact, gluten is substituted by other ingredients such as fibers and fats in order to mimic the sensory and technological characteristics of the protein, but providing different nutritional characteristics. Therefore, the consumption of these rendered products could be another source of dietary imbalance. Thus, inadequate macronutrient distribution has been reported, characterized by high fat and protein and low carbohydrate content, as well as a micronutrient intake deficit [3,9,10,11,12,13]. 

Nutritional advice should be essential among people with celiac disease. Nevertheless, dieticians/nutritionists working with this collective can make little more than approximations when evaluating a gluten-free diet (GFD). Most current databases of dietary software do not include nutritional information of gluten-free products (GFP), so that the diet of people with celiac disease cannot be precisely designed and their nutrient intake is miscalculated.

Our research group, GLUTEN3S, has been working in gluten detection in food, beverage, and parapharmacy products for many years [14]. Concurrently, GLUTEN3S has studied GFD quality in order to detect most common mistakes, the reasons for them, and strategies for their solution [6,15,16]. As we inferred from our results, the dietary pattern is one of the main factors explaining above mentioned macro and micronutrient imbalances. In general, the consumption of cereals and vegetables is very low, intake of fruit scarce and that of meat and derivatives excessive. This shows the importance of implementing specific nutritional guidelines for people on a GFD. Furthermore, the role of GFP in the diet should not be forgotten. After demonstrating that nutritional composition of GFP differed from that of their gluten-containing analogues [9], this has been a matter of concern for our group [17]. For all of these reasons, the aim of the present work was to create a software containing the nutritional composition of GFPs that could help people with celiac disease and their clinicians in the design and evaluation of a balanced gluten-free diet (GFD).

## 2. Materials and Methods 

### 2.1. Database Preparation

Before software design, a food composition database was developed. The nutritional composition of conventional foods was obtained from BEDCA net [18], the database of the Spanish Agency for Food Consumption and Security (AESAN). This database contains more than 2000 generic foods with their complete nutritional composition (energy, macro and micronutrient content). 

In order to complete this database with gluten-free rendered products, first of all, and in collaboration with the Basque Country Celiac Society (EZE), a thorough selection of the foodstuffs most consumed by people with CD was carried out, such as, breads, flours, pasta, biscuits, pastry, etc. In total, more than 700 GFP from different commercial brands (>50) were selected. Their nutritional composition, which included energy content, proteins, carbohydrates, sugars, fats, saturated fats, fiber, and salt, was taken from food labels. The brand of each product was included in the database. Both AESAN and the trade companies of GFP gave their consent for the inclusion of the data of their products in this new database.

### 2.2. Software Programming

Once the database had been completed, the software for gluten-free diet design (GlutenFreeDiet, http://www.ehu.eus/dieta-singluten/) was programmed using WordPress, PHP, and MySQL for server site and using jQuery for customer site, which are the programming languages. This technology is the most used among content managers and has a wide market share so that it offers stability [19].

### 2.3. Reference Values and Questionnaires

The software was designed to provide an easy and intuitive program for entering dietary, anthropometric, and biochemical data and turning them into interesting information regarding dietary balance and nutritional status. Dietary data is collected following the schema of a 24 h food-recall and food group consumption frequency [20,21]. Energy and nutrient intake or food group consumption suitability is calculated according to the AESAN guidelines [22,23]. What is more, other adapted questionnaires were also included in order to measure the presence of gastrointestinal symptoms [24], the adherence to gluten-free diet [25], or quality of life of people with celiac disease [26]. Basal metabolism, energy expenditure, and Body Mass Index were calculated and evaluated according to World Health Organization (WHO) formulae, while biochemical data follow Basque Health system references. 

### 2.4. Software Validity Evaluation

First of all, to check that software calculations were appropriate, two dietitians compared energy and macronutrient content (carbohydrates, lipids, proteins, and fiber) of normocaloric, hypercaloric, hyperproteic, and hyperlipidic gluten-containing diets (*n* = 4 each) calculated by hand [27] and through the software.

Secondly, in order to demonstrate the necessity of incorporating GFP composition into databases, 35 24 h recalls from adults and 35 from children with CD were evaluated. Carbohydrates, lipids, protein, fiber, and energy intake were evaluated, and those results compared to the calculation of the same diets substituting GFP with their gluten-containing counterparts. This evaluation was performed separately for adults and children because different nutritional recommendations are described for each collective [6,15,16]. Adult participants were recruited from a cohort between 2011 and 2012 from three regions in the north of Spain (Araba, Gipuzkoa, and Bizkaia). Mean age (mean ± standard deviation) was 47 ± 12 and 45 ± 14 years and median age (range:years) was 23–71:45 and 34–61:45 for women and men respectively. Children and adolescents with CD were from 4 to 15 years of age (mean ± standard deviation 8.4 ± 3.9, and median 8.5) and were recruited during 2011 and 2013 from the same three regions of the Basque Country. All patients were members of EZE, with a confirmation of CD diagnosis (intestinal biopsy and/or serological test). The inclusion criteria were the follow-up of a GFD for at least one year and to be in remission from clinical symptoms. Exclusion criteria included patients suffering from other pathologies apart from CD that made them follow a special diet (diabetes, allergies, etc.) or patients suffering from serious disease (such as cancer) [6,15,16]. All participants received verbal and written information about the nature and purpose of the survey, and all gave their written consent for their involvement in the study. Both studies were approved by the Ethical Committee of the University of The Basque Country (CEISH/76/2011 and CEISH/194M/2013).

Finally, diets were recalculated with Alimentación y Salud^®^ software (with no GFP in its database; Granada, Spain), and results in carbohydrates, lipids, protein, fiber, and energy intake were compared to those obtained by GlutenFreeDiet software.

### 2.5. App Design

The mobile app was developed with the Flutter open source development kit that favors agile development to produce intuitive and easy-to-use applications. It also allows one to generate versions for Android and iOS and it is compiled natively so it provides good performance at runtime, against other current libraries.

### 2.6. Statistical Analysis

Statistical analyses of results were performed by using the IBM SPSS statistical program, version 23 (IBM Inc., Armonk, NY, USA). Normality in the distribution was assessed by the Kolmogorov–Smirnov test, and homogeneity by Levene’s test. Statistical differences of dietary results calculated trough different software or by hand were analyzed with Wilcoxon test. *p* values < 0.05 were accepted as significant.

## 3. Results

### 3.1. Software for GFD Evaluation and Design

**Figure 1:**
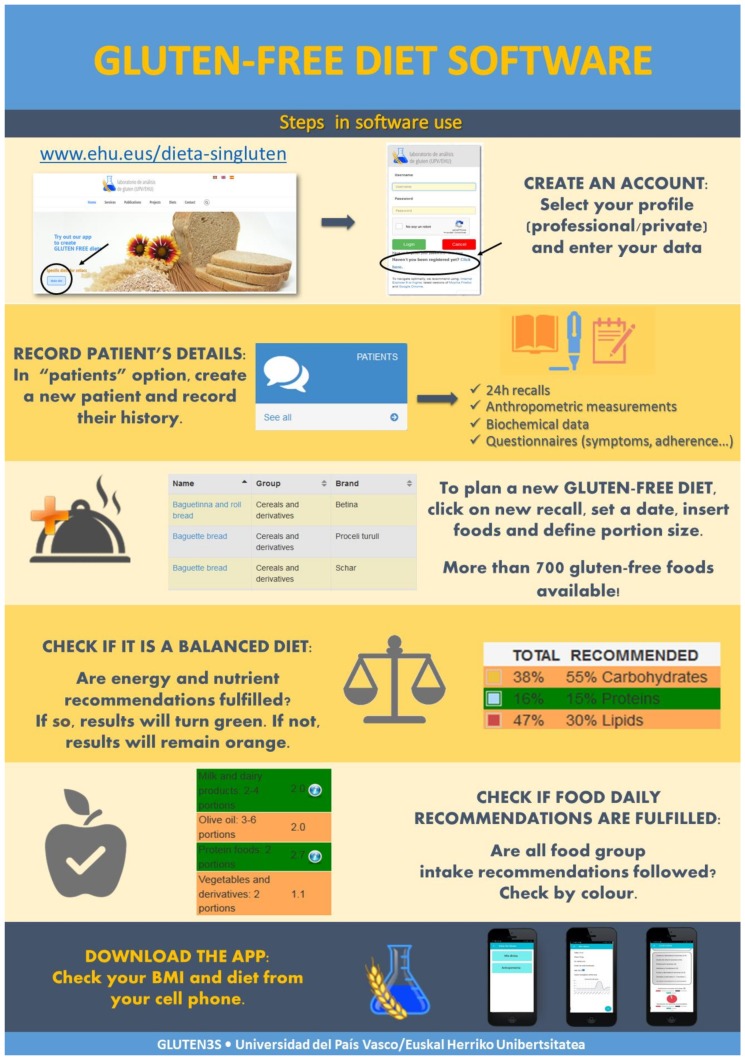
Scheme explaining software use and functionality.

Figure 1 shows software functionality graphically. This software allows users to register as a health professional or as an individual user (for people on the GFD). All users have to create an account and wait until the profile is activated. At the professional profile, the application presents the opportunity to introduce data about patients and their diets, biochemical analysis, or anthropometric results for a complete evaluation of their nutritional status. After that, patient’s diet can be evaluated, or new and personalized diets can be designed. GFP’s brands and nutritional composition can be observed while working on the diet (Supplementary Figure S1). Once finished, nutritional balance and fulfillment of food group consumption are shown with easy visual tables and figures.

The software can be also conceived as a self-care tool. Colored charts and tables were designed in order to give nutritional education and advice. Patients can realize the reasons for their dietary imbalance. Nutrient intake or food group ingestion fulfillment appears in green. When exceeding or not achieving recommendations, colors turn orange. Moreover, the recommended intake is detailed in all tables and charts. 

An explanatory tutorial about software use is available on our group’s web page (www.ehu.eus/es/web/laboratorio_gluten/home).

The GlutenFreeDiet software also gives the chance to measure other factors that affect patients’ health status, such as the presence of gastrointestinal symptoms or the adherence to GFD. The first one includes 15 questions related to abdominal pain or distension, heartburn, or defecation frequency with seven possible degrees of discomfort. The second one is composed of four questions with dichotomic responses in order to analyze the dietary behavior.

### 3.2. App Version of the Software

In addition to the software, an application for cell phones and tablets was designed, which can be downloaded from the software (Figure 1) but it is still not available in the store for Android and iOS. The app keeps the same useful and intuitive design of the software, as well as being accessible any time and place. 

### 3.3. Software Evaluation

No differences were obtained after calculating normocaloric, hypercaloric, hyperproteic, and hyperlipidic diets by hand and by the software (Table 1). By contrast, when 24 h recalls of celiac children and adults were calculated by GlutenFreeDiet software selecting (1) GFP or (2) replacing them with gluten-containing homologues, some differences arose: higher energy, carbohydrate and fat (in adults), and lower protein intake were observed when using the specific GFP database (Table 2). Finally, when GlutenFreeDiet software was compared to another commercial program, significant differences were observed in energy, protein, carbohydrate (in children) and fat amounts (Table 2).

## 4. Discussion

It is well known that the only possible treatment for gluten related disorders is a strict lifelong GFD. However, following a secure and balanced GFD is not an easy task. The approach to a healthy GFD should be done from two perspectives: (i) the strict withdrawal of gluten, and (ii) the appropriate balance of the diet to avoid deficiencies and optimize metabolic processes. 

Despite an apparent correct follow-up of the diet, there is a high percentage of celiac and/or gluten-sensitive population that does not respond sufficiently, thus histological damage or intestinal symptoms persist [28]. Some authors attributed it to a possible low adherence or to dietary transgressions [29,30,31]. They stated that this low adherence could be related to unintended gluten ingestion and lack of training and to an unawareness of the importance of not consuming even traces of gluten. Therefore, self-perception of adherence could lead to an overestimation of GFD real adherence. Moreover, a systematic review performed by Hall et al. found that strict adherence ranged from 42% to 91% of celiac people [32]. This reflects the difficulties celiac people must face when taking dietary decisions, and highlights their need for specific training.

As stated in the introduction, people on a GFD frequently choose, apart from naturally gluten-free foods, specific gluten-free rendered products. In the case of children, it has been published that GFP provides about a quarter of the total energy intake [16,33]. It has been said that, in general terms, diets including GFP result in less balance, since they have more fat and lower amounts of carbohydrates, proteins, and fiber [7,9,34,35,36]. These nutritional deviations could be due to several reasons, such as the ingredients used to improve the texture and/or palatability of GFP and the qualitative and quantitative nutritional differences in cereals of a GFD [2,9,36]. Moreover, they reveal the necessity of suitable food composition databases and information tools in order to properly measure and evaluate GFD.

As far as we know, examples of dietary management software available on the worldwide market do not contain the nutritional composition of a large number of GFP. They focus on dietary plan designs for healthy and/or sporty people giving personal advice and coaching. Moreover, most of them are not freely available. Some of these programs are also useful for therapeutic dietary plans, and include alternatives for people suffering from diseases such as diabetes, hypercholesterolemia, or hypertension. However, none of them contain enough GFP in their databases to evaluate or design varied GFD, so are not suitable for celiac people. Even though a great deal of information about gluten-free cooking, gluten detection in labels, or gluten-free alternatives and safe restaurants is available online, there are no software programs for GFD planning.

Evaluation of GlutenFreeDiet software demonstrated first the correct functionality of the software. Secondly, the differences between GF and GC products composition were reflected in the diets. Thus, results comparing GF and GC diets underlined the importance of evaluating GFD considering the nutritional composition of GFP. Even if scientific effort in this field has been important recently, this has not transferred across to clinical practice, where different GFP composition is not being taken into account. Thus, it could be supposed that dietary advice from health professionals could be inaccurate. Indeed, the comparison between GlutenFreeDiet software and a commercial one showed the importance of using proper dietary programs when GFD are analyzed.

Another important matter of concern for people working in the field of celiac disease is the maintenance of suitable nutritional status in these patients. Many celiac people suffer from malnutrition before they are diagnosed [37,38,39]. This situation could be thought to be remedied when patients begin a GFD. However, there is some controversy about the normalization of nutritional deficiencies and anthropometric measurements, which could continue to be unsuitable [6,38,40,41]. Even more, there is evidence that complete normalization of duodenal mucosa is rare [28]. This software gives the chance to measure and monitor these and other parameters, such as biochemical data, the presence of symptoms or the adherence to the diet, which are indicators of the nutritional status and quality of life of celiac people. 

Apart from the dietitian or clinician care, self-care is a very important aspect to approach in the case of people on a GFD. Specialized nutritional and dietary advice has been demonstrated to be very useful in order to enhance it and to promote adherence [42]. Moreover, it has been suggested that it should be included in the follow-up of celiac people [3]. Currently, there are several information sources that celiac people use to take decisions about their diets. The most used come from association support, from other celiac people with experience, from cookbooks and the internet [43]. In fact, e-Health is gaining interest within health institutions, as internet and technology can reach users easily and rapidly, with a wide range of contents and attractive formats. Here, we present free and practical software, not only useful for professionals working in the field of celiac disease, but also for celiac people. Apart from dietary programming, it gives nutritional education through messages, colored charts, tables, and documentation that can be freely downloaded. Following these recommendations, the patient can improve his or her dietary mistakes, vary diet, and finally reach a dietary balance.

Taking all the above into consideration, GlutenFreeDiet is a useful software whose strengths are the large number of GFP collected in its database and its configuration as an open free platform with an educative character. Likewise, it has been designed as a tool for transferring knowledge, working as a meeting point between patients and clinicians. Nevertheless, it is fair to say that it is a new start-up and so it is important to track its functionality and user satisfaction in order to promote its continuous improvement. For this reason, the program has been designed to be versatile, allowing future enhancements such as user suggestions, new GFPs, graphic design, and so on. In fact, in the near future the software will add some other information, which will be very useful for GFD management, such as sugar, FODMAPs (Fermentable Oligosaccharides, Disaccharides, Monosaccharides and Polyols) content (from either GF or gluten-containing food), as well as the micronutrient content of the GFP (based on ingredients’ contribution), which is also information lacking in the common databases. 

Moreover, we expect to create a virtual space with visual and attractive information about gluten-free and healthy diets. Teaching-Learning Sequence about celiac disease and GFD for kids, infographics, comments on the latest advances in gluten related disorderss and material from any kind of educational activities performed by the GLUTEN3S will be available for any interested person. In addition, links to other reliable sources of information (research groups, celiac associations, etc.) will be included. As a goal, all the material that can help this collective to improve their self-caring and quality of life will be freely available.

## 5. Conclusions

Online tools have the potential to reach a large number of people in the privacy of their homes. They are therefore a good way to bring closer scientific information to increase knowledge about healthy food choices and self-care. GlutenFreeDiet software could represent an easy, trustworthy, attractive, and interactive tool with an exclusive GFP composition database for training not only gluten-sensitive users but also professionals in the field of GFD design. Overall, this interactive tool aims to improve the quality of life of the former by improving their capacity for self-care and the accuracy of dietary advice from the latter.

## Figures and Tables

**Table 1:** Energy, carbohydrates, protein, fat, and fiber content of normocaloric, hypercaloric, hyperlipidic, and hyperproteic gluten-containing diets.

	Energy (kcal)			Proteins (g)			Lipids (g)			Carbohydrates (g)			Fiber (g)		
	By Hand	Software	*p*	By Hand	Software	*p*	By Hand	Software	*p*	By Hand	Software	*p*	By Hand	Software	*p*
Hypercaloric	4808.8 ± 102.6	4817.0 ± 167.2	NS	179.7 ± 12.1	179.0 ± 10.0	NS	190.9 ± 11.6	196.1 ± 15.3	NS	628.0 ± 10.2	589.1 ± 7.7	NS	66.0 ± 14.0	62.7 ± 15.3	NS
Normocaloric	2549.9 ± 80.0	2469.2 ± 37.0	NS	99.6 ± 8.4	103.3 ± 7.4	NS	92.4 ± 3.9	89.0 ± 5.4	NS	339.9 ± 12.7	310.6 ± 7.1	NS	32.7 ± 4.6	34.2 ± 5.1	NS
Hyperproteic	2435.3 ± 263.3	2531.6 ± 338.2	NS	129.8 ± 15.3	131.9 ± 12.6	NS	92.0 ± 7.8	96.9 ± 13.2	NS	281.8 ± 40.0	279.5 ± 47.1	NS	36.6 ± 4.8	36.3 ± 8.1	NS
Hyperlipidic	2743.1 ± 52.9	2777.8 ± 83.2	NS	102.8 ± 12.8	103.2 ± 12.3	NS	140.4 ± 13.7	145.9 ± 20.3	NS	283.5 ± 29.4	268.5 ± 36.9	NS	36.9 ± 8.8	39.4 ± 8.6	NS

Notes: Values are means ± SD. SD, standard deviation.

**Table 2:** Gluten-free diet (GFD) energy and nutrient content of a cohort of celiac people calculated by GlutenFreeDiet software (GFsoft) and comparison to (1) data obtained by substituting gluten-free products (GFPs) with gluten containing analogues using GlutenFreeDiet software (GCsoft) and (2) data obtained by another commercial software that does not contain GFP in its database (CGAyS).

	GF_soft_	GC_soft_	*p_1_*	GC_AyS_	*p_2_*
Energy (kcal)					
Adults	1653.4 ± 471.4	1627.9 ± 481.0	0.08	1619.7 ± 480.6	0.002
Children	1817.3 ± 354.0	1809.7 ± 360.1	NS	1776.9 ± 360.6	0.009
Proteins (g)					
Adults	84.4 ± 23.5	87.5 ± 23.8	0.001	86.4 ± 23.5	0.014
Children	72.5 ± 14.2	77.8 ± 15.0	<0.001	78.2 ± 12.9	<0.001
Lipids (g)					
Adults	69.3 ± 32.0	71.7 ± 32.7	0.04	67.3 ± 31.0	<0.001
Children	82.8 ± 28.2	81.9 ± 27.6	NS	76.0 ± 26.0	0.02
Carbohydrates (g)					
Adults	157.8 ± 59.8	154.3 ± 55.1	0.02	164.9 ± 59.5	NS
Children	201.2 ± 56.0	197.0 ± 54.1	0.01	209.3 ± 57.4	0.015
Fiber (g)					
Adults	15.6 ± 5.8	15.8 ± 5.6	NS	15.6 ± 6.7	NS
Children	14.9 ± 4.5	14.2 ± 3.8	NS	14.6 ± 4.0	NS

Notes: Values are means ± SD. SD, standard deviation. GF_soft_: Gluten-free calculated by GlutenFreeDiet software; GC_soft_: Gluten containing calculated by GlutenFreeDiet software; GC_AyS_: Gluten containing calculated by AlimentaciónySalud commercial software. *p*, statistical significance; *p_1:_* GF_soft_ vs. GC_soft_; *p_2:_* GF_soft_ vs. GC_AyS_. NS, not significant.

## Supplementary Materials

The following are available online at https://www.mdpi.com/2072-6643/11/10/2505/s1, Figure S1: Steps to design a gluten-free diet with the software.

## References

[1] Lionetti E., Catassi C. (2011). New clues in celiac disease epidemiology, pathogenesis, clinical manifestations, and treatment. Int. Rev. Immunol..

[2] Simón E., Larrechi I., Churruca I., Lasa A., Bustamante M.A., Navarro V., Fernández-Gil M.P., Miranda J. (2017). Nutritional and Analytical Approaches of Gluten-Free Diet in Celiac Disease.

[3] Vici G., Belli L., Biondi M., Polzonetti V. (2016). Gluten free diet and nutrient deficiencies: A review. Clin. Nutr..

[4] Bardella M.T., Fredella C., Prampolini L., Molteni N., Giunta A.M., Bianchi P.A. (2000). Body composition and dietary intakes in adult celiac disease patients consuming a strict gluten-free diet. Am. J. Clin. Nutr..

[5] Capristo E., Malandrino N., Farnetti S., Mingrone G., Leggio L., Addolorato G., Gasbarrini G. (2009). Increased serum high-density lipoprotein-cholesterol concentration in celiac disease after gluten-free diet treatment correlates with body fat stores. J. Clin. Gastroenterol..

[6] Churruca I., Miranda J., Lasa A., Bustamante M., Larretxi I., Simon E. (2015). Analysis of Body Composition and Food Habits of Spanish Celiac Women. Nutrients.

[7] Martin J., Geisel T., Maresch C., Krieger K., Stein J. (2013). Inadequate nutrient intake in patients with celiac disease: Results from a german dietary survey. Digestion.

[8] Theethira T.G., Dennis M. (2015). Celiac disease and the gluten-free diet: Consequences and recommendations for improvement. Dig. Dis..

[9] Miranda J., Lasa A., Bustamante M.A., Churruca I., Simon E. (2014). Nutritional Differences Between a Gluten-free Diet and a Diet Containing Equivalent Products with Gluten. Plant Food Hum. Nutr..

[10] Penagini F., Dilillo D., Meneghin F., Mameli C., Fabiano V., Zuccotti G.V. (2013). Gluten-free diet in children: An approach to a nutritionally adequate and balanced diet. Nutrients.

[11] Thompson T. (1999). Thiamin, riboflavin, and niacin contents of the gluten-free diet: Is there cause for concern?. J. Am. Diet. Assoc..

[12] Cornicelli M., Saba M., Machello N., Silano M., Neuhold S. (2018). Nutritional composition of gluten-free food versus regular food sold in the Italian market. Dig. Liver Dis..

[13] Kulai T., Rashid M. (2014). Assessment of Nutritional Adequacy of Packaged Gluten-free Food Products. Can. J. Diet. Pract. Res..

[14] Bustamante M.Á., Fernández-Gil M.P., Churruca I., Miranda J., Lasa A., Navarro V., Simon E. (2017). Evolution of Gluten Content in Cereal-Based Gluten-Free Products: An Overview from 1998 to 2016. Nutrients.

[15] González T., Larretxi I., Vitoria J.C., Castaño L., Simón E., Churruca I., Navarro V., Lasa A. (2018). Celiac Male’s Gluten-Free Diet Profile: Comparison to that of the Control Population and Celiac Women. Nutrients.

[16] Larretxi I., Simon E., Benjumea L., Miranda J., Bustamante M.A., Lasa A., Eizaguirre F.J., Churruca I. (2019). Gluten-free-rendered products contribute to imbalanced diets in children and adolescents with celiac disease. Eur. J. Nutr..

[17] Larretxi I., Txurruka I., Navarro V., Lasa A., Bustamante M., Fernández-Gil M.D.P., Simón E., Miranda J. (2019). Micronutrient Analysis of Gluten-Free Products: Their Low Content Is not Involved in Gluten-Free Diet Imbalance in a Cohort of Celiac Children and Adolescent. Foods.

[18] AESAN Base de Datos Española de Composición de Alimentos (BEDCA) v 1.0. http://www.bedca.net/.

[19] W3Techs. https://w3techs.com/.

[20] Bel-Serrat S., Mouratidou T., Pala V., Huybrechts I., Börnhorst C., Fernández-Alvira J.M., Hadjigeorgiou C., Eiben G., Hebestreit A., Lissner L. (2014). Relative validity of the Children’s Eating Habits Questionnaire-food frequency section among young European children: The IDEFICS Study. Public Health Nutr..

[21] Rodriguez V., Elbusto A., Alberdi M., De la Presa A., Gómez F., Portillo M., Churruca I. (2014). New pre-coded food record form validation. Rev. Esp. Nutr. Hum. Diet..

[22] Dapcich V., Salvador G., Ribas L., Perez C., Aranceta J., Serra L. (2004). Guía de Alimentación Saludable.

[23] FESNAD (2010). Dietary reference intakes (DRI) for spanish population. Act. Diet..

[24] Laurikka P., Salmi T., Collin P., Huhtala H., Mäki M., Kaukinen K., Kurppa K. (2016). Gastrointestinal Symptoms in Celiac Disease Patients on a Long-Term Gluten-Free Diet. Nutrients.

[25] Morisky D.E., Green L.W., Levine D.M. (1986). Concurrent and predictive validity of a self-reported measure of medication adherence. Med. Care.

[26] Van Doorn R.K., Winkler L.M., Zwinderman K.H., Mearin M.L., Koopman H.M. (2008). CDDUX: A disease-specific health-related quality-of-life questionnaire for children with celiac disease. J. Pediatric Gastroenterol. Nutr..

[27] Mataix J., Mataix J. (2009). Tablas de Composición de Alimentos.

[28] Lanzini A., Lanzarotto F., Villanacci V., Mora A., Bertolazzi S., Turini D., Carella G., Malagoli A., Ferrante G., Cesana B.M. (2009). Complete recovery of intestinal mucosa occurs very rarely in adult coeliac patients despite adherence to gluten-free diet. Aliment. Pharmacol. Ther..

[29] Dewar D.H., Donnelly S.C., McLaughlin S.D., Johnson M.W., Ellis H.J., Ciclitira P.J. (2012). Celiac disease: Management of persistent symptoms in patients on a gluten-free diet. World J. Gastroenterol..

[30] Lebwohl B., Sanders D.S., Green P.H.R. (2018). Coeliac disease. Lancet.

[31] Silvester J.A., Weiten D., Graff L.A., Walker J.R., Duerksen D.R. (2016). Is it gluten-free? Relationship between self-reported gluten-free diet adherence and knowledge of gluten content of foods. Nutrition.

[32] Hall N.J., Rubin G., Charnock A. (2009). Systematic review: Adherence to a gluten-free diet in adult patients with coeliac disease. Aliment. Pharmacol. Ther..

[33] Zuccotti G., Fabiano V., Dilillo D., Picca M., Cravidi C., Brambilla P. (2013). Intakes of nutrients in Italian children with celiac disease and the role of commercially available gluten-free products. J. Hum. Nutr. Diet..

[34] Kinsey L., Burden S.T., Bannerman E. (2008). A dietary survey to determine if patients with coeliac disease are meeting current healthy eating guidelines and how their diet compares to that of the British general population. Eur. J. Clin. Nutr..

[35] Thompson T., Dennis M., Higgins L.A., Lee A.R., Sharrett M.K. (2005). Gluten-free diet survey: Are Americans with coeliac disease consuming recommended amounts of fibre, iron, calcium and grain foods?. J. Hum. Nutr. Diet..

[36] Shepherd S.J., Gibson P.R. (2013). Nutritional inadequacies of the gluten-free diet in both recently-diagnosed and long-term patients with coeliac disease. J. Hum. Nutr. Diet..

[37] Capristo E., Addolorato G., Mingrone G., De Gaetano A., Greco A.V., Tataranni P.A., Gasbarrini G. (2000). Changes in body composition, substrate oxidation, and resting metabolic rate in adult celiac disease patients after a 1-y gluten-free diet treatment. Am. J. Clin. Nutr..

[38] Corazza G.R., Di Sario A., Sacco G., Zoli G., Treggiari E.A., Brusco G., Gasbarrini G. (1994). Subclinical coeliac disease: An anthropometric assessment. J. Intern. Med..

[39] Capristo E., Mingrone G., Addolorato G., Greco A.V., Corazza G.R., Gasbarrini G. (1997). Differences in Metabolic Variables between Adult Coeliac Patients at Diagnosis and Patients on a Gluten-Free Diet. Scand. J. Gastroenterol..

[40] Kemppainen T.A., Kosma V.M., Janatuinen E.K., Julkunen R.J., Pikkarainen P.H., Uusitupa M.I. (1998). Nutritional status of newly diagnosed celiac disease patients before and after the institution of a celiac disease diet--association with the grade of mucosal villous atrophy. Am. J. Clin. Nutr..

[41] Smecuol E., Gonzalez D., Mautalen C., Siccardi A., Cataldi M., Niveloni S., Mazure R., Vazquez H., Pedreira S., Soifer G. (1997). Longitudinal study on the effect of treatment on body composition and anthropometry of celiac disease patients. Am. J. Gastroenterol..

[42] Sainsbury K., Mullan B., Sharpe L. (2013). A Randomized Controlled Trial of an Online Intervention to Improve Gluten-Free Diet Adherence in Celiac Disease. Am. J. Gastroenterol..

[43] Zarkadas M., Dubois S., MacIsaac K., Cantin I., Rashid M., Roberts K.C., La Vieille S., Godefroy S., Pulido O.M. (2013). Living with coeliac disease and a gluten-free diet: A Canadian perspective. J. Hum. Nutr. Diet..

