# Supplementary material for: New Software for Gluten-Free Diet Evaluation and Nutritional Education
Source: Nutrients. 2019 Oct 17;11(10):2505. doi: 10.3390/nu11102505 (PMC6836287; doi:10.3390/nu11102505)
Supplement: Supplementary file 1 [file nutrients-11-02505-s001.pdf]

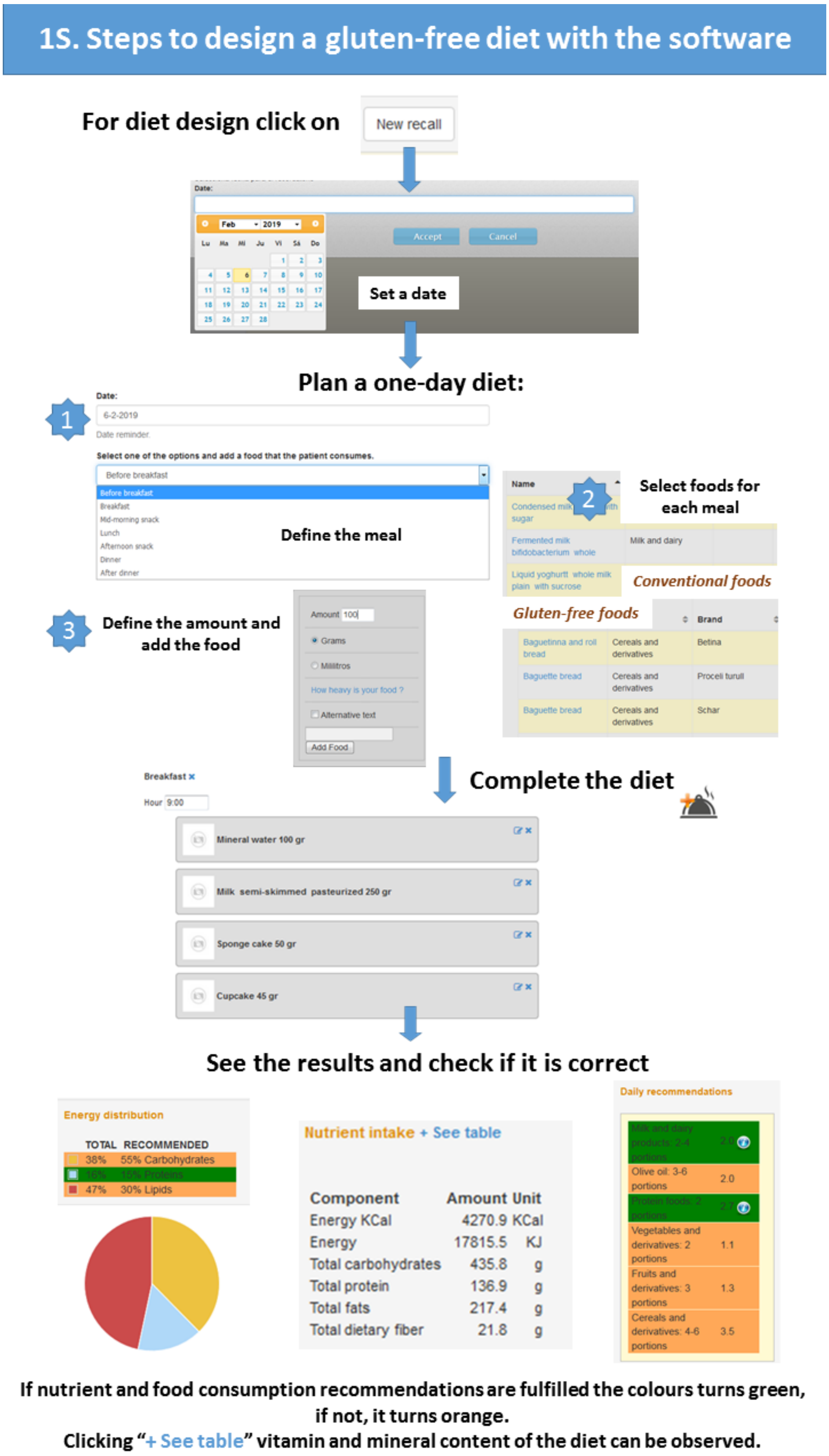

Figure 1. Steps to design a gluten-free diet with the software.
